# LCP external fixation - External application of an internal fixator: two cases and a review of the literature

**DOI:** 10.1186/1749-799X-5-19

**Published:** 2010-03-20

**Authors:** Colin Yi-Loong Woon, Merng-Koon Wong, Tet-Sen Howe

**Affiliations:** 1 Department of Orthopaedic Surgery, Singapore General Hospital,169608, Singapore

## Abstract

The locking compression plate (LCP) is an angle-stable fixator intended for intracorporeal application. In selected cases, it can be applied externally in an extracorporeal location to function as a monolateral external fixator. We describe one patient with Schatzker V tibial plateau fracture and one patient with Gustillo IIIB open tibia shaft fracture treated initially with traditional external fixation for whom exchange fixation with externally applied LCPs was performed. The first case went on to bony union while the second case required bone grafting for delayed union. Both patients found that the LCP external fixators facilitated mobilization and were more manageable and aesthetically acceptable than traditional bar-Schanz pin fixators.

## Introduction

Plate external fixation is not a new concept. While it has been described in the management of open fractures [1-3], nonunion [1-4], septic arthritis [2] and even as an adjunct in distraction osteogenesis [5] (Table 1), it is still deemed unconventional and does not enjoy the same place in classical textbooks as other methods of fracture fixation.

**Table 1:** Comparison of Reports of Plate External Fixation

Author	Year of Publication	Number of Patients	Indications for Plate External Fixation	Bones involved	Implant type	Temporary or Definitive	Average Duration on LCP external fixation	Infection (%)	Nonunion (%)
Kloen [4]	2009	4	Infected nonunion	1 clavicle, 3 tibia	3.5 or 4.5 mm LCP	3 temporary, 1 definitive	4 months (2 - 6)	0	0

Apivatthakakul and Savanpanich [5]	2007	1	Bone transport*	Tibia	4.5 mm broad LCP	Definitive	5 months†	0	0

Kerkhoffs et al [2]	2003	31	9 open fractures, 18 infected nonunion, 3 septic arthritis‡, 1 infected pathological fracture	12 forearm, 2 clavicle, 4 humerus, 6 tibia, 4 elbow, 1 olecranon, 1 femur, 1 shoulder	DCP with nuts and washers	Definitive	12 weeks (2 - 23)	2/23 (9) §	4/31 (1)

Ramotowski and Granowski [3]	1991	1212	850 fractures	191 femur, 493 tibia, 45 humerus, 64 radius, 52 ulna, 5 others||	Zespol system	Definitive	18 weeks	NM	44/850 (5)**
					
			445 nonunions	106 femur, 245 tibia, 40 humerus, 22 radius, 31 ulna, 1 other||		Definitive	21 weeks	1 (4) ¶	27/445 (6) ¶

Marti and van der Werken [1]	1991	12	4 open fractures, 7 infected nonunion, 1 septic arthritis	7 forearm, 1 clavicle, 1 humerus, 2 tibia, 1 shoulder	DCP with nuts and washers	Definitive	NM	2/12 (17) **	2/12 (17) **

LCP, locking compression plate; DCP, dynamic compression plate; NM, not mentioned

* Together with Wagner limb lengthening device

† On LCP external fixator alone, after removal of Wagner device

‡ Plate external fixation for arthrodesis in septic arthritis (2 elbow, 1 shoulder).

§Persistent infection in 2 cases of septic arthritis.

|| Only on tibia and ulna bones were Zespol applied externally, for a total of 545 tibia and ulna fractures, and 276 tibia and ulna nonunions.

¶Including cases treated with paraosseous and subcutaneous Zespol application.

** Infected nonunion (1 clavicle, 1 humerus).

Understandably, the design of implants of old, such as the Zespol implant (Mikromed Sp. zo.o., Dabrowa Górnicza, Poland) [3], or dynamic compression plates (DCPs; Synthes Inc, Paoli, PA) coupled with multiple nuts and washers [1,2], may have dissuaded surgeons who may have been otherwise more receptive to this technique. With the advent of anatomically-contoured locking-head plates with fewer moving parts, there has been a resurgence of interest in this technique, as evidenced by the publications that have surfaced over the last decade. It may thus be timely to consider the merits of this novel technique and examine the situations where it may be indicated.

In this report, we describe our early experience with use of this technique. While the first case progressed uneventfully to bony union, the second required secondary bone grafting and later internal fixation with a locking compression plate (LCP), serving to reinforce that as with all novel procedures, there is a steep learning curve and cases should be carefully selected. We further review the published literature and explore the caveats and pitfalls of applying this novel method of external fixation.

Both patients were informed that data concerning their cases would be submitted for publication.

## Surgical Technique

For both our cases, exchange external fixation with an LCP was performed. Initial steps are similar to exchange application of a traditional external fixator. After positioning the patient on a radiolucent operating table, excisional debridement and pulsed lavage is performed under general anesthesia and with tourniquet control. If an external fixator is already in place, attention is paid to thorough cleansing of the external fixator prior to its removal at this stage.

**Figure 1:**
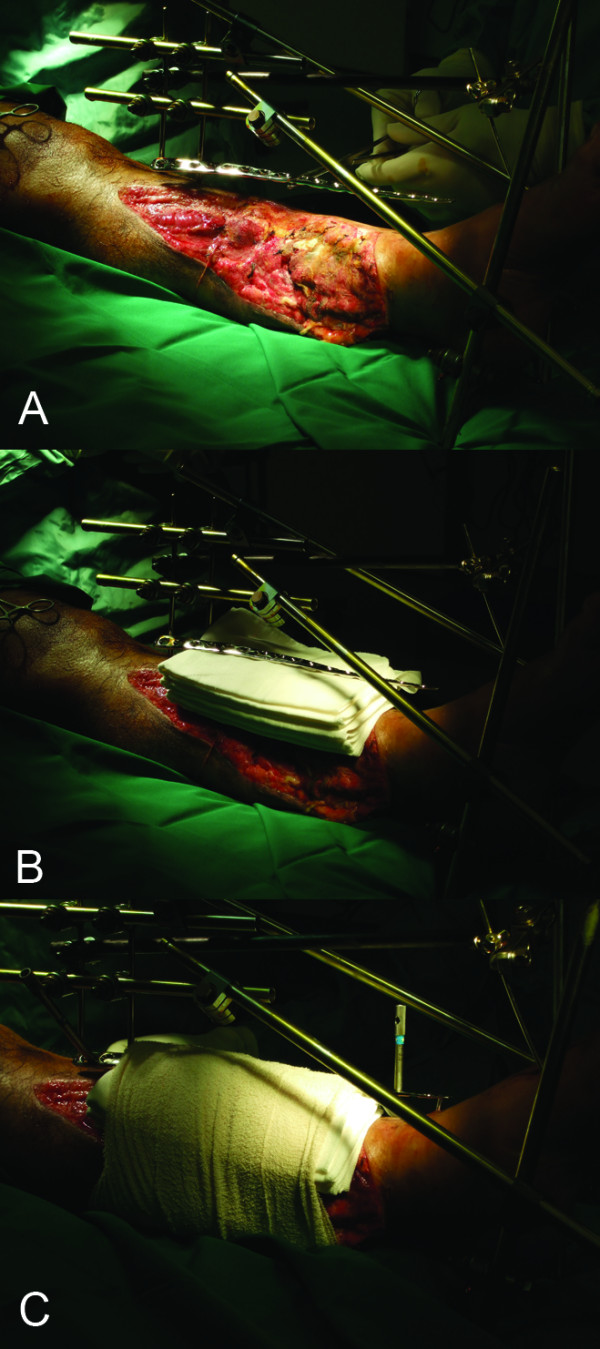
**a - Selection of a LCP of appropriate length to span the fracture fragments**. The LCP may be contoured or twisted to facilitate soft tissue coverage. b - A stack of folded towels functions as a spacer of uniform thickness. c - The spacer is secured to limb with elastic bandage. The most proximal and distal screw holes are drilled first. The bar-Schanz pin construct provides the reduction and is left in situ until completion.

An LCP of sufficient length to span the fracture fragments is chosen (Fig 1a), with the aim of engaging at least 4 to 6 cortices in each major fragment, taking care to avoid implanting screws at the fracture site. The principle of symmetry [6,7] (same screw type and number, and distance separating screws on each side of the fracture) is observed [8,9]. The plate may be contoured to facilitate later soft tissue coverage or to address bone fragments where bone purchase is greatest. The chosen LCP is placed over the desired application site, separated from the skin surface by a spacer of uniform thickness, such as a stack of evenly folded towels (Fig 1b). This spacer is then firmly bandaged to the limb with an elastic bandage (Fig 1c), taking care to avoid covering the most proximal and distal holes intended for initial screw placement. Satisfactory plate placement is then confirmed fluroscopically. Successive holes are drilled over locking drill-guides through stab incisions where the overlying soft tissue envelope is intact and screws are placed. The entire construct is then reassessed fluoroscopically. When alignment is deemed satisfactory, the screw sites and the remaining soft tissue defect are dressed in the usual fashion.

LCP external fixation is best applied to subcutaneous bones such as the tibia, clavicle and ulna to minimize screw-site problems associated with soft tissue motion. Standard pin-care protocols apply. We use gentle compressive dressings between the plate and skin with regular saline cleansing at each dressing change. Patients provide their own screw-site care with soap and water during daily personal hygiene routines upon discharge.

## Case 1

**Figure 2:**
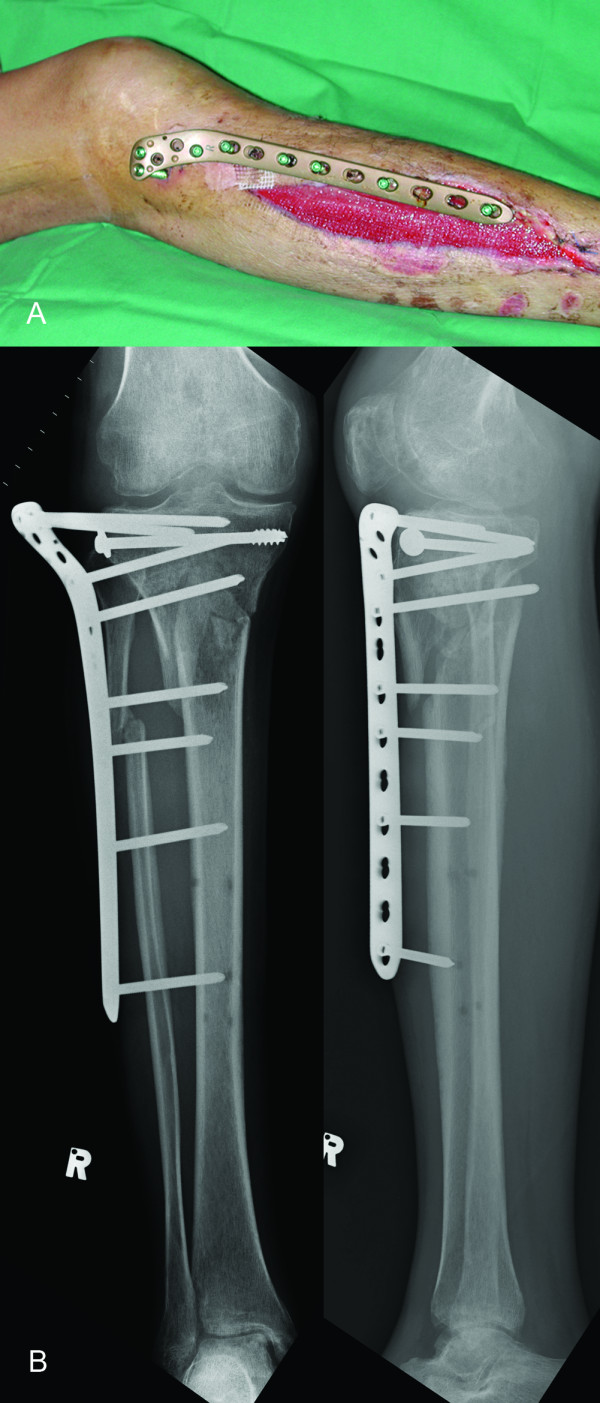
**a - External appearance of proximal tibia LCP applied as an external fixator**. b - Postoperative radiograph showing proximal tibia LCP external fixation.

A 54-year old male motorcyclist was involved in a motor-vehicle accident with a car. He sustained closed Schatzker V [10] right tibial plateau and fibula shaft fractures. On presentation, there was marked swelling of the right leg, with blistering of the overlying skin and severe pain on passive dorsiflexion of the ankle. He was diagnosed with compartment syndrome of the right leg and underwent emergency two-incision fasciotomy and external fixation within nine hours of presentation. Intravenous antibiotics were continued in the perioperative period. In the first postoperative week, he underwent two further surgical debridements and dressing changes owing to dressing staining with malodorous, greenish discharge from both fasciotomy wounds. The presence of continuous wound discharge made internal fixation hazardous at this point. Ten days after the initial operation, the traditional external fixator was removed and a 9-hole 4.5 mm proximal tibia LCP (Synthes Inc, Paoli, PA) plate was applied as an external fixator (Figs 2a &2b). Delayed primary closure was performed for both fasciotomy wounds. He progressed to full weightbearing at four months. Eight months after the initial LCP external fixation, radiographs revealed bony union with acceptable alignment. There were no complications such as screw loosening or soft tissue complications. The LCP external fixator was removed in clinic under local anesthesia.

## Case 2

**Figure 3:**
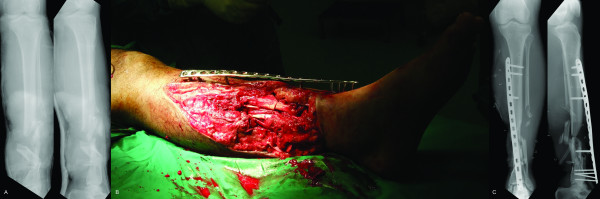
**a - Comminuted Gustillo-Anderson IIIB open diaphyseal fractures of the right tibia and fibula**. b - Exchange external fixation performed with LCP contoured to facilitate soft tissue coverage. c - Postoperative radiograph of LCP external fixation.

A 38-year old male motorcyclist was involved in a motor-vehicle accident in which he was flung from his vehicle. He sustained open fractures of the left tibia and fibula shafts (Gustillo-Anderson grade IIIB) (Fig 3a) [11]. Wound debridement and application of an external fixator was performed on admission. After 72 hours, he underwent re-look and repeat debridement. Five days after the initial injury, vacuum-assisted closure dressing (VAC; Kinetic Concepts, Inc, San Antonio, Tex.) was applied. The following day, the external fixator was exchanged for an 18-hole 4.5 mm combination LCP (Synthes Inc, Paoli, PA) (Figs 3b &3c). Plastic surgical consult was obtained to best site the fixator where it would not be in the way of later soft tissue coverage. A gentle twist was imparted to the plate to improve distal bone fragment purchase. He underwent 11 further debridements owing to wound colonization with *Acinetobacter baumannii*, and later, methicillin-resistant *Staphylococcus aureus *(MRSA). Soft tissue resurfacing was finally achieved with a combination of split-thickness skin graft and free dermal graft. He was discharged one month after the original operation. At six months, fibula pro tibia grafting was performed for delayed union resulting from bone loss at the fracture site. The fibula graft was compressed and secured to the tibia with two cortical screws, with the LCP external fixator left untouched. While the LCP external fixator was in place, there were no signs of local screw-site sepsis or screw loosening.

A third operation was performed three months later because of unacceptable valgus malalignment and dorsal angulation. This involved removal of the external fixation and replacement with an internally placed LCP pilon plate 2.7/3.5 (Synthes Inc, Paoli, PA), with more screws in the distal fragment, coupled with iliac crest bone grafting. Bony union was noted four months later, during which time he had progressed to full weightbearing with a walking aid.

## Discussion

Traditional external fixator constructs (bar and half-pin, ring, hybrid or newer modular designs) are employed either for temporary damage control or as definitive fixation [1] in high-grade open fractures to provide stability while avoiding superinfection of an internal fixation device. However, traditional frames are often bulky and ambulating with a lower limb fixator frame in-situ is awkward. Some patients are self-conscious of these fixators and find them less aesthetically acceptable, especially when more visible locations such as the ulna and clavicle are involved.

Conceptually, the angle-stable locking compression plate (LCP) is an internally placed unibody, monolateral fixator. Although designed for epiperiosteal application, increasing the plate-to-bone distance for locations with a pronounced muscle sleeve results in submuscular placement, desirable where comminution is present to bridge fragments while preserving vascularity. For subcutaneous bones such as tibia, ulna or clavicle, increasing the plate-to-bone distance lifts the LCP into an extra-corporeal location, while preserving its inherent characteristics of flexibility (long-span) and stability (locked-screw) [12]. This concept has been previously elaborated upon by Ramotowski and Granowski [3], who defined the possible depths of plate fixation as paraosseous, subcutaneous and external osteosynthesis for femur, humerus and tibia or ulna respectively.

## Pitfalls and Caveats

While the first case was uncomplicated, our second case went into nonunion, requiring conversion to internal fixation. A nonunion rate of 5-17% has been noted by other authors versed in this technique (Table 1) [1-5] comparable with rates of nonunion (up to 20%) [13] in traditional external fixation. Nonunion in Case 2 can be attributed to the nature of LCP application and characteristics of the LCP that make it stand apart from traditional external fixation. First, while traditional external fixation employs introduction of half pins prior to cross-bar connection, LCP external fixation requires drilling and screw placement through predetermined plate holes while the plate is suspended above bone. During plate application, both plate and bone fragment can move independently, making accurate screw placement difficult as small shifts at the plate translate to great deviations at the level of bone. Second, with a single screw in place, plate movement is confined to rotation in one plane and once two or more screws are placed, alterations in plate position are no longer possible. Third, unlike the more forgiving traditional fixator, the monoaxial nature of the locking-head screw trajectory reduces the ability to compensate for imperfect placement, making it mandatory that anatomical reduction be achieved prior to placement of the first screw. Should adjustment be required following application, it may be necessary to abandon either the drilled bone hole or the selected plate hole. Fourth, the small space beneath the plate makes it difficult to apply vascularized soft tissue cover. Flap inset on top of a plate might lead to tension on the pedicle and pose problems for later hardware removal. To site the fixator away from the open wound in Case 2 in anticipation of later soft tissue coverage, the LCP was twisted to achieve a proximal-anteromedial, distal-anterior plate siting (Fig 3b) instead of a fully anteromedial placement. Another strategy to facilitate dressing changes and soft tissue coverage involves plate twisting or incorporating a "wave" design [4]. This must be done with caution so as to avoid disruption of plate threads, thereby precluding screw placement. Fifth, while traditional constructs can be strengthened by stacking cross-bars, this is not possible for LCP external fixation. A more rigid construct can be created by reducing the moment arm with a thinner spacer (fewer folded towels during plate application), increasing overall screw number, placing screws closer to the fracture, and increasing the distance between screws in each screw group [6,8,14]. Alternatively, Kloen's strategy of double fixation (two LCPs on the tibia) may be attempted to surmount this problem [4]. Sixth, screw placement is absolutely limited to the available screw holes of the chosen plate. Valgus drift in Case 2 may have been potentiated by inadequate screw purchase in the small distal fragment and compounded by screw loosening in increasingly osteopenic bone.

Certain considerations must be borne in mind when concentric bones such as humerus and femur are involved. Bicortical engagement may not always be possible owing to limitations on available screw length. If so, this must be compensated by use of more unicortical screws, bearing in mind that unicortical configurations have 50% less rigidity than bicortical purchase [6,8].

Finally, additional cost is incurred if initial fixation is with a conventional external fixator (such as in both above cases). This is not the case if LCP external fixation is used primarily [2], although we have no experience with this.

## Advantages

We agree with Kloen [4] that the LCP has certain advantages when used in this manner. First, the LCP fixator imparts a lower profile than a traditional fixator and can be concealed under clothing, making it more acceptable to patients [2,4,5]. Second, hardware removal can be performed in an outpatient setting under local anesthesia (Case 1). Third, the LCP fixator imparts a less conspicuous radiographic silhouette compared with traditional fixators (Figs 2b and 3c).

Other theoretical advantages remain to be tested experimentally. First, small amounts of axial micromotion may reduce stress-shielding of the fracture site. Load-sharing during weight bearing may stimulate the developing callus until bony union [12]. Second, "controlled destiffening" or dynamization by removing screws closest to the fracture site is possible, allowing some measure of control to the load-sharing process [12].

## Conclusion

LCP external fixation is an unconventional alternative to traditional external fixation. While it may be of benefit in carefully selected cases of fractures and nonunions, it is not without its own unique set of complications. Close clinical and radiological follow-up is necessary to detect fixation failure. In this event, the surgeon should consider converting to rigid internal fixation. Biomechanical studies may be of benefit in comparing the biomechanical characteristics of this construct with traditional fixator designs.

## Consent

Written informed consent was obtained from the patient for publication of this case report and any accompanying images. A copy of the written consent is available for review by the Editor-in-Chief of this journal.

## Competing interests

The authors declare that they have no competing interests.

## Authors' contributions

Dr CYLW conceived and wrote the paper. Dr MKW and Dr TSH were the surgeons of the two patients and revised the manuscript critically for intellectual content. All the authors read and approved the final manuscript.
